# Application of a validated prostate MRI deep learning system to independent same-vendor multi-institutional data: demonstration of transferability

**DOI:** 10.1007/s00330-023-09882-9

**Published:** 2023-07-28

**Authors:** Nils Netzer, Carolin Eith, Oliver Bethge, Thomas Hielscher, Constantin Schwab, Albrecht Stenzinger, Regula Gnirs, Heinz-Peter Schlemmer, Klaus H. Maier-Hein, Lars Schimmöller, David Bonekamp

**Affiliations:** 1 Division of Radiology, German Cancer Research Center (DKFZ), Im Neuenheimer Feld 280, 69120 Heidelberg, Germany; 2 Heidelberg University Medical School, Heidelberg, Germany; 3 Medical Faculty, Department of Diagnostic and Interventional Radiology, University Dusseldorf, D-40225 Dusseldorf, Germany; 4 Division of Biostatistics, German Cancer Research Center (DKFZ), Heidelberg, Germany; 5 Institute of Pathology, University of Heidelberg Medical Center, Heidelberg, Germany; 6 German Cancer Consortium (DKTK), Heidelberg, Germany; 7 National Center for Tumor Diseases (NCT) Heidelberg, Heidelberg, Germany; 8 Medical Image Computing, German Cancer Research Center (DKFZ), Heidelberg, Germany; 9 Pattern Analysis and Learning Group, Department of Radiation Oncology, Heidelberg University Hospital, Heidelberg, Germany

**Keywords:** Prostatic neoplasms, Magnetic resonance imaging, Deep learning, Validation study

## Abstract

**Objectives:**

To evaluate a fully automatic deep learning system to detect and segment clinically significant prostate cancer (csPCa) on same-vendor prostate MRI from two different institutions not contributing to training of the system.

**Materials and methods:**

In this retrospective study, a previously bi-institutionally validated deep learning system (UNETM) was applied to bi-parametric prostate MRI data from one external institution (A), a PI-RADS distribution-matched internal cohort (B), and a csPCa stratified subset of single-institution external public challenge data (C). csPCa was defined as ISUP Grade Group ≥ 2 determined from combined targeted and extended systematic MRI/transrectal US-fusion biopsy. Performance of UNETM was evaluated by comparing ROC AUC and specificity at typical PI-RADS sensitivity levels. Lesion-level analysis between UNETM segmentations and radiologist-delineated segmentations was performed using Dice coefficient, free-response operating characteristic (FROC), and weighted alternative (waFROC). The influence of using different diffusion sequences was analyzed in cohort A.

**Results:**

In 250/250/140 exams in cohorts A/B/C, differences in ROC AUC were insignificant with 0.80 (95% CI: 0.74–0.85)/0.87 (95% CI: 0.83–0.92)/0.82 (95% CI: 0.75–0.89). At sensitivities of 95% and 90%, UNETM achieved specificity of 30%/50% in A, 44%/71% in B, and 43%/49% in C, respectively. Dice coefficient of UNETM and radiologist-delineated lesions was 0.36 in A and 0.49 in B. The waFROC AUC was 0.67 (95% CI: 0.60–0.83) in A and 0.7 (95% CI: 0.64–0.78) in B. UNETM performed marginally better on readout-segmented than on single-shot echo-planar-imaging.

**Conclusion:**

For same-vendor examinations, deep learning provided comparable discrimination of csPCa and non-csPCa lesions and examinations between local and two independent external data sets, demonstrating the applicability of the system to institutions not participating in model training.

**Clinical relevance statement:**

A previously bi-institutionally validated fully automatic deep learning system maintained acceptable exam-level diagnostic performance in two independent external data sets, indicating the potential of deploying AI models without retraining or fine-tuning, and corroborating evidence that AI models extract a substantial amount of transferable domain knowledge about MRI-based prostate cancer assessment.

**Key Points:**

*• A previously bi-institutionally validated fully automatic deep learning system maintained acceptable exam-level diagnostic performance in two independent external data sets.*

*• Lesion detection performance and segmentation congruence was similar on the institutional and an external data set, as measured by the weighted alternative FROC AUC and Dice coefficient.*

*• Although the system generalized to two external institutions without re-training, achieving expected sensitivity and specificity levels using the deep learning system requires probability thresholds to be adjusted, underlining the importance of institution-specific calibration and quality control.*

## Introduction

Artificial intelligence has demonstrated potential to support radiological assessment of prostate MRI by providing fully automatic detection and segmentation of suspicious lesions; i.e., similar diagnostic performance to clinical PI-RADS [1] assessment has been shown in retrospective studies during primary development and validation [2], within simulated deployment [3] and with the use of deep transfer learning [4]. Recently, the performance of a U-Net [5]-based deep learning (DL) model [6] (referred to as UNETM) trained on 806 single-vendor multi-scanner (two 3.0 Tesla and one 1.5 Tesla scanners) examinations from a single institution was not improved on the institutional test set of 682 examinations or within the PROSTATEx challenge [7, 8] (140 test exams) when training was extended to a bi-institutional data set which included 204 additional PROSTATEx training examinations. Rather, increasing the training set size was the most important determinant of performance compared to scanner heterogeneity or bi-institutional training data. These findings highlighted generalization capability and transferability of prostate MRI DL models, as apparently the domain-specific knowledge—at least in a single-vendor setting—can be well extracted from large albeit institution- or scanner-specific data sets. Such findings motivate evaluation of models on further external data without re-training the model, under the assumption that protocol and scanner differences do not render the CNN unable to extract pertinent information from the images. However, based on machine learning assumptions and prior study results, it cannot be generally assumed that the performance of models trained within one institution remains consistent when presented with data from external institutions which did not contribute to the training [9–11]. Thus, such instances require further investigation. While the PROSTATEx dataset provides same-vendor data from a different institution, it is limited in terms of characterization as not all details of the patient characteristics and biopsy are available.

The aim of this study was to evaluate UNETM performance without re-training on a clinically well-characterized dataset acquired at a collaborating institution constructed by combining a balanced number of consecutive csPCa-positive and csPCa-negative bi-parametric prostate MRI exams and on a balanced subset of the PROSTATEx challenge data not previously used for training, thus providing two independent external data sets to study success of extraction of csPCa-specific knowledge in different practice settings. In addition, diffusion-weighted MRI sequences were varied to assess the influence of different protocols.

## Material and methods

This retrospective analysis was performed in men who received mpMRI and MRI–transrectal or transperineal US (MRI/TRUS) fusion–guided biopsy at the participating institutions. All men had suspicion for clinically significant prostate cancer (csPCa) based on prostate-specific antigen (PSA) elevation or clinical examination and had no prior history of csPCa. Balanced data sets were constructed as follows.

In Düsseldorf (site A), the institutional ethics committee approved the study (2020-1038) with all patients having given written informed consent for data transfer to Heidelberg for analysis. Beginning in 01/2018, 25 consecutive examinations resulting in csPCa diagnoses by in-house targeted plus systematic MRI/US fusion-guided biopsy (UroNAV, Invivo, Philips) were identified and supplemented by the first 25 examinations not resulting in csPCa diagnosis. These cases were fully annotated at voxel-level by matching pathology reports to clinically called lesions and performing manual delineation to form data set A-F. Similarly, beginning in 01/2017, 100 additional consecutive MR examinations positive for csPCa were identified and supplemented with the first 100 csPCa-negative examinations in that period and annotated only at patient-level according to the maximum Gleason score seen in biopsy. Together with data set A-F, these examinations form data set A-1.

In Heidelberg (site B), the institutional ethics committee approved the study (S-164/2019) with a waiver for written informed consent for local data analysis. To form data set B, a subset of 250 examinations dating from 10/2017 to 7/2020 from a previously published test set [6] and meeting the inclusion and exclusion criteria for this study was extracted to mirror the PI-RADS distribution found in data from site A. One hundred seventy-seven of these examinations were annotated at voxel-level in addition to patient-level (data set B-F).

From the PROSTATEx training set (site C), all csPCa-positive examinations were extracted, annotated at voxel-level, and supplemented by an equal number of randomly selected csPCa-negative examinations (data set C, 140 examinations). The balanced design was chosen to allow focus on discriminative ability of the UNETM model independent of disease prevalence and PI-RADS suspicion at different institutions.

Men from sites A and B were included if they met the following criteria: (a) presentation for institutional mpMRI of the prostate during the inclusion period; and (b) same institutional MRI/TRUS-fusion biopsy performed subsequently. Exclusion criteria were (a) history of treatment for prostate cancer (antihormonal therapy, radiation therapy, focal therapy, prostatectomy); (b) biopsy within 2 months prior to MRI or time to biopsy after MRI exceeding 6 months; (c) incomplete sequences or severe MRI artifacts or incomplete biopsy. csPCa was defined as International Society of Urological Pathology grade ≥ 2 [12].

### MR imaging

T2-weighted, diffusion-weighted (DWI), and dynamic contrast-enhanced MRI from the collaborating institutions were acquired on six different MRI systems (Heidelberg: Prisma 3 .0T, BiographMR 3.0T, Aera 1.5T; Düsseldorf: Prisma 3.0 T, Tim Trio 3.0 T, Skyra 3.0 T; Siemens Healthineers), with imaging performed based on PI-RADS v2.1 guidelines, by using the standard multichannel body coil and integrated spine phased-array coil. PROSTATEx training set exams were acquired on two different scanners (Tim Trio 3.0 T, Skyra 3.0 T; Siemens Healthineers) and were available from the public challenge website. The prostate MRI protocols used in the three cohorts are given in Table 1. At site A, a total of two different diffusion sequences were available over the study period, albeit not all for each patient, with some patients receiving more than one diffusion sequence. For primary analysis, the readout-segmented echo-planar imaging (rs-EPI) sequence was selected first, followed by single-shot echo-planar imaging (ss-EPI), by availability, to additionally challenge the analysis by a diffusion sequence different from the one used during training. For assessment of the resulting influence of diffusion sequence type, a second version of data set A-1, set A-2, was defined by reversing the diffusion sequence preference order for comparison of receiver operating characteristics (ROC) area under the curve (AUC) and diagnostic operating points.

**Table 1:** MR protocols

Scanner	Field strength (T)	Sequence	*b*-values	Acquisition plane	TE (ms)	TR (ms)	In-plane resolution (mm)	Slice thickness (mm)	FOV x (mm)	FOV y (mm)	FOV %
Cohort A											
Siemens Prisma (*N* = 136)	3 T	T2w	–	tra	101–105	3710–4060	0.5–0.51	3	130–160	130–160	100
		DWI	0, 500, 1000 + 1800 calc	tra	50	4540–5810	1.43	3	200	200	100
Siemens Skyra (*N* = 39)	3 T	T2w	–	tra	84	8200–8269	0.51–0.625	3	130–160	130–160	100
		DWI	0, 500, 100 + 1600 calc	tra	55	6200–7615	1.79	3	200	200	100
Siemens Tim Trio (*N* = 75)	3 T	T2w	–	tra	117	10,630	0.51–0.664	3.3	130–170	130–170	100
		DWI	0, 500, 1000 + 0, 1400	tra	90	4600–4700	1.471–1.5	3	200–204	199–204	100
Cohort B											
Siemens Prisma (*N* = 246)	3 T	T2w	–	tra	105–145	3710–8672	0.3125–0.51	3	130–200	130–200	100
		DWI	0 or 50, 500, 1000, 1500	tra	48–71	4000–4600	2	3	208	280	74.29–100
Siemens Biograph mMR (*N* = 2)	3 T	T2w	–	tra	145–146	4000–8000	0.26–0.313	3–3.3	199–200	199–200	100
		DWI	0 or 50, 500, 1000, 1500	tra	67–81	4900–7600	1–1.1	3	208–210	280	74.29–75
Siemens Aera (*N* = 2)	1.5 T	T2w	–	tra	100–116	3790–5710	0.2815–0.42	3	159–180	180	100
		DWI	0 or 50, 500, 1000, 1500	tra	68	5100–5300	1.17–2.33	3-4	242–279	279	86.67–100
Cohort C											
Siemens Tim Trio (*N* = 5)	3 T	T2w	–	tra	103	4480–5306	0.5625	3	180	180	100
		DWI	50, 400, 800	tra	64	2500	2	4	256	212	82.8125
Siemens Skyra (*N* = 135)	3 T	T2w	–	tra	104	5660–6840	0.5	3–3.15	192	192	100
		DWI	50, 400, 800	tra	63	2700–3200	2	3	168	256	65.625

### Data annotation

Annotation of data set A-1 was performed at site A. For data set A-1, targeted and systematic biopsy histopathological information and MR lesion information was recorded. Additionally, using the Medical Imaging Interaction Toolkit [13, 14], three-dimensional regions of interest were drawn manually by one operator (C.E.) to segment each individual MR visible lesion under supervision of a board-certified radiologist and prostate MRI expert with 11 years of experience in prostate MRI (L.S.) on 50 examinations from data set A-F. Data were then sent to site B for combined analysis. Data from site B are a subset of a previously published test set [6] with annotations and histopathology information already available for analysis. PROSTATEx training data have been segmented at site B previously under supervision of a board-certified radiologist (D.B.) based on coordinate information provided by the challenge.

### Histopathological assessment

At site A, all patients received targeted MRI/US fusion-guided biopsy of MR-visible lesions with additional 12-core transrectal systematic biopsy (UroNAV, Invivo, Philips).

At site B, all patients received transperineal TRUS/MRI fusion biopsies according to the Ginsburg protocol [15], for a median of 25 systematic biopsies. In addition, each MR visible lesion was targeted using a median of 5 cores. Histopathological assessment was performed under the supervision of an experienced genitourinary pathologist (A.S. with 15 years of experience).

### Fully automated deep learning–based assessment

Bi-parametric data of all examinations, including axial T2-weighted (T2w), the DWI image with the highest available b-value, and ADC maps from diffusion-weighted imaging (DWI, either ss-EPI or rs-EPI) were input into a previously developed fully automated deep learning pipeline [6] based on the U-Net architecture [5] implemented using the nnUNet framework [16]. Briefly, an ensemble of five 3D and five 2D U-Nets configured by standard nnUNet heuristics (batch size of two, six downsampling blocks, patch-based inference) were used to predict sPC on stacked, quantatively normalized ADC maps and z-scored T2w and DWI. The utilized model (referred to here as UNETM) has been previously trained on 806 single-vendor multi-scanner (two 3.0 Tesla and one 1.5 Tesla scanners) examinations from site B and exhibited the overall best performance compared to models trained with less data, single-scanner models, and, importantly, also a model including PROSTATEx training cases in training. Thus, the selected UNETM model has been evaluated only on the PROSTATEx *test* set but has been trained solely using MR data from site A, leaving the PROSTATEx *training* set as an independent external test set for model evaluation. The model outputs a three-dimensional softmax map indicating a number between 0 and 1 for each pixel in the image given in the same spatial orientation and resolution as the T2 sequence. The higher the value in the softmax map, the higher the suspicion for csPCa. To obtain patient-level predictions, the highest value in each exam was used. The pipeline automatically performs prostate segmentation, elastic co-registration of the two DWI-image series to the T2w series, and cropping as part of the analysis. No further user interaction was required.

### Lesion-level assessment

For lesion segmentation overlap evaluation, the three-dimensional softmax map was thresholded at a value required to reach patient-level sensitivity of 95% and postprocessed using connected component analysis and speckle removal. Lesions were considered jointly detected with radiologists if they showed any overlap. Lesions were considered successfully detected if at least 10% of their voxels were segmented by the model. Performance was evaluated against the standard of clinical lesion segmentations with csPCa diagnosed either in corresponding targeted biopsy cores or in overlapping sextant systematic biopsy cores (systematic-enhanced lesion ground truth), or in the latter extended by lesion-free however systematic biopsy-positive sextants (positive-sextant extended ground truth, fusion ground truth). In the latter approach, sextant segmentations with no clinical lesion overlap that were positive for csPCa were added to the set of lesion segmentations. This avoids mistakenly counting false positive model predictions in direct vicinity of csPCa or in remote systematic biopsy-only detected csPC regions and provides an important additional view on the possible spatial mappings of the available histopathological information.

### Statistical analysis

Patient-level ROC analysis was used to compare and plot the performance of fully automated UNETM assessment for data set A, data set B, and data set C. Differences in discrimination ability were assessed using the Delong test [17]. Operating points at ≥ 95% and ≥ 90% sensitivity were determined for each data set by selecting the corresponding UNETM threshold and resulting specificity compared using Fisher’s exact test [18] or McNemar’s test [19]. All statistical comparisons were corrected for multiple comparisons using Holm’s method [20] and used a significance level of *p* < 0.05. PI-RADS operating points at ≥ 3 and ≥ 4 thresholds are given for orientation, which are however considered less comparable as these originate from clinical assessment in a different context and not from assessment on the cohorts examined here and thus may carry over institutional prevalence effects, other than the CNN assessment which is newly calculated for all three cohorts. Detailed lesion-level analysis was performed using free-response receiver operating characteristic (FROC, and weighted alternative (waFROC)) analysis, using the R package RJafroc [21]. waFROC analysis was chosen to avoid cases with many lesions inordinately influencing results [22] and to better reflect the importance of MRI evaluation avoiding biopsy altogether [23] by considering the fraction of histopathologically negative cases with at least one MRI lesion. Statistical analyses were implemented in R (R version 4.0.2; R Foundation for Statistical Computing) [24].

## Results

### Study sample

Table 1 shows the MR protocols used for examinations. Table 2 shows the demographic data and patient characteristics of all 640 examinations included in the study. Cohort A included 250 men with a median age of 69 (IQR 63–76) and PSA density of 0.17 (IQR 0.12–0.24). Cohort B included 250 examinations with a median age of 65 (IQR 58–70) and PSA density of 0.16 (IQR 0.12–0.27). Cohort C included 140 men with a median age 65 (IQR 60–69). The ratio of csPCa to non-csPCa in cohorts A and C was 1:1, and 0.87:1 for cohort B. Data set A-1 consists of 250 exams primarily using rs-EPI. A-2 consists of the same exams but uses ss-EPI preferentially. Data sets A-F and B-F are fully voxel-level annotated subsets of A-1 and B, for which lesion-wise Gleason scores are available. In data set C (PROSTATEx), all sPC harboring lesions were annotated according to challenge provided coordinates.

**Table 2:** Demographic and clinical characteristics of the analyzed cohorts. Values given for cohort C encompass Gleason Score 6 or lower and 7a or higher, respectively

Cohort	A	B	C
	*n* = 250	*n* = 250	*n* = 140
Age (years) Median (IQR)	69 (63–76)	64 (57–70)	65 (60–69)
Overall PI-RADS Score (*n* (%)) 2 3 4 5	7 (3%) 53 (21%) 105 (42%) 85 (34%)	7 (3%) 53 (21%) 105 (42%) 85 (34%)	– – – –
Per-patient maximum Gleason Score (*n* (%)) no PC 6 (3+3) 7a (3+4) 7b (4+3) 8 (4+4) 9 (4+5) 9 (5+4) 10 (5+5)	88 (35%) 38 (15%) 65 (26%) 27 (11%) 23 (9%) 8 (3%) 0 (0%) 1 (0%)	100 (40%) 34 (14%) 65 (26%) 15 (6%) 15 (6%) 16 (6%) 3 (1%) 2 (1%)	– 70 (50%) 70 (50%) – – – – –
PSA (ng/mL) median (IQR) PSA density median (IQR)	7.8 (5.9–11.6) 0.17 (0.12–0.24)	8.0 (5.9–11.7) 0.16 (0.12–0.27)	– –
Biopsy distribution per patient (*n* (%)) Biopsy-naïve Previously biopsied Active surveillance	152 (61%) 95 (38%) 3 (1%)	170 (68%) 42 (17%) 38 (15%)	– – –

### Ability to detect csPCa

**Fig. 1:**
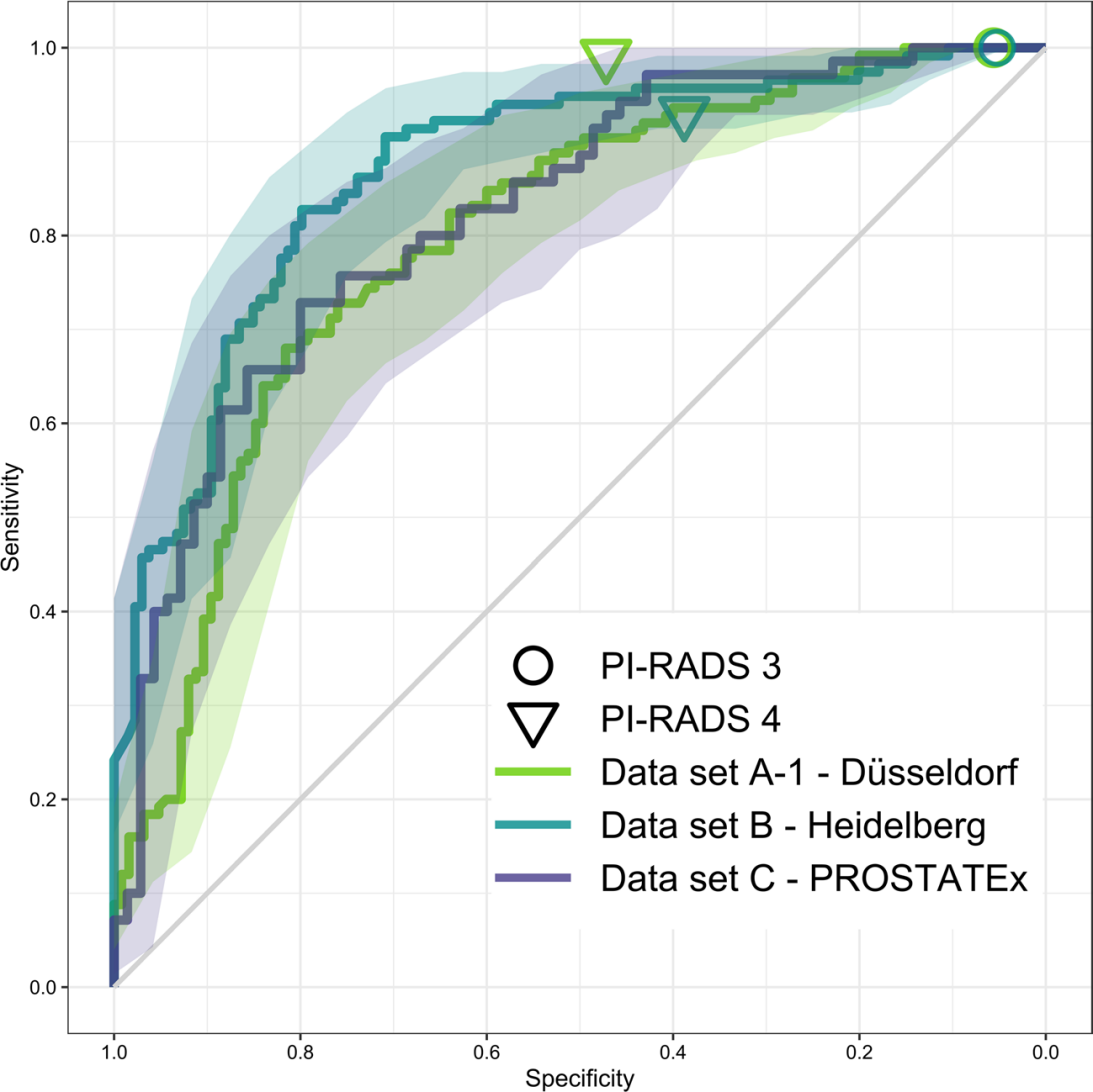
Receiver operating characteristics (ROC) curves and 95% confidence intervals for patient-level analysis of UNETM performance in data set A-1 (green), B (cyan) and C (purple), with clinical PI-RADS performance as measured by csPCa detection shown as circles (PI-RADS 3; green in site A Düsseldorf, cyan in site B Heidelberg) and triangles (PI-RADS 4)

Patient-level ROC AUC of fully automated UNETM assessment was 0.80 in data set A-1, 0.87 in data set B, and 0.82 in data set C, with Delong test indicating no statistical difference (all *p* ≥ 0.135) (Fig. 1). At chosen clinically useful sensitivity thresholds of ≥ 95%, specificity was 30% [37/125] in data set A, 44% [59/134] in data set B, and 43% [30/70] in data set C. At fixed sensitivity of ≥ 90%, specificity was 50% [62/125] in data set A-1, 71% [95/134] in data set B, and 49% [34/70] in data set C. Fisher’s exact test indicated no statistical difference between specificity in all data sets at the ≥ 95% sensitivity threshold (*p* > 0.08) and a significantly lower specificity in datasets A-1 and C in comparison to data set B at the ≥ 90% sensitivity threshold (*p* < 0.011) (Table 3). For comparison, radiologist sensitivity was 100% at PI-RADS 3 with specificities of 6% [7/125] and 5% [7/134] in data sets A-1 and B, respectively. Sensitivity at PI-RADS 4 was 99% in data set A-1 and 93% in data set B with corresponding specificities of 47% [59/125] and 39% [52/134]. Radiologist sensitivity and specificity however are not comparable to the clinical performance of PI-RADS at both institutions, as the balanced study design necessarily affects these measurements. They are provided for comparison to DL performance only.

**Table 3:** Specificity comparisons at fixed sensitivity (Fisher’s exact test)

Sensitivity	Specificity	Data set 1	Data set 2	Adj. *p*
≥ 0.90	0.71/0.50	B	A-1	0.003
≥ 0.90	0.71/0.49	B	C	0.011
≥ 0.90	0.49/0.5	C	A-1	1
≥ 0.95	0.44/0.30	B	A-1	0.082
≥ 0.95	0.44/0.43	B	C	1
≥ 0.95	0.43/0.30	C	A-1	0.25

### Lesion-level assessment

**Fig. 2:**
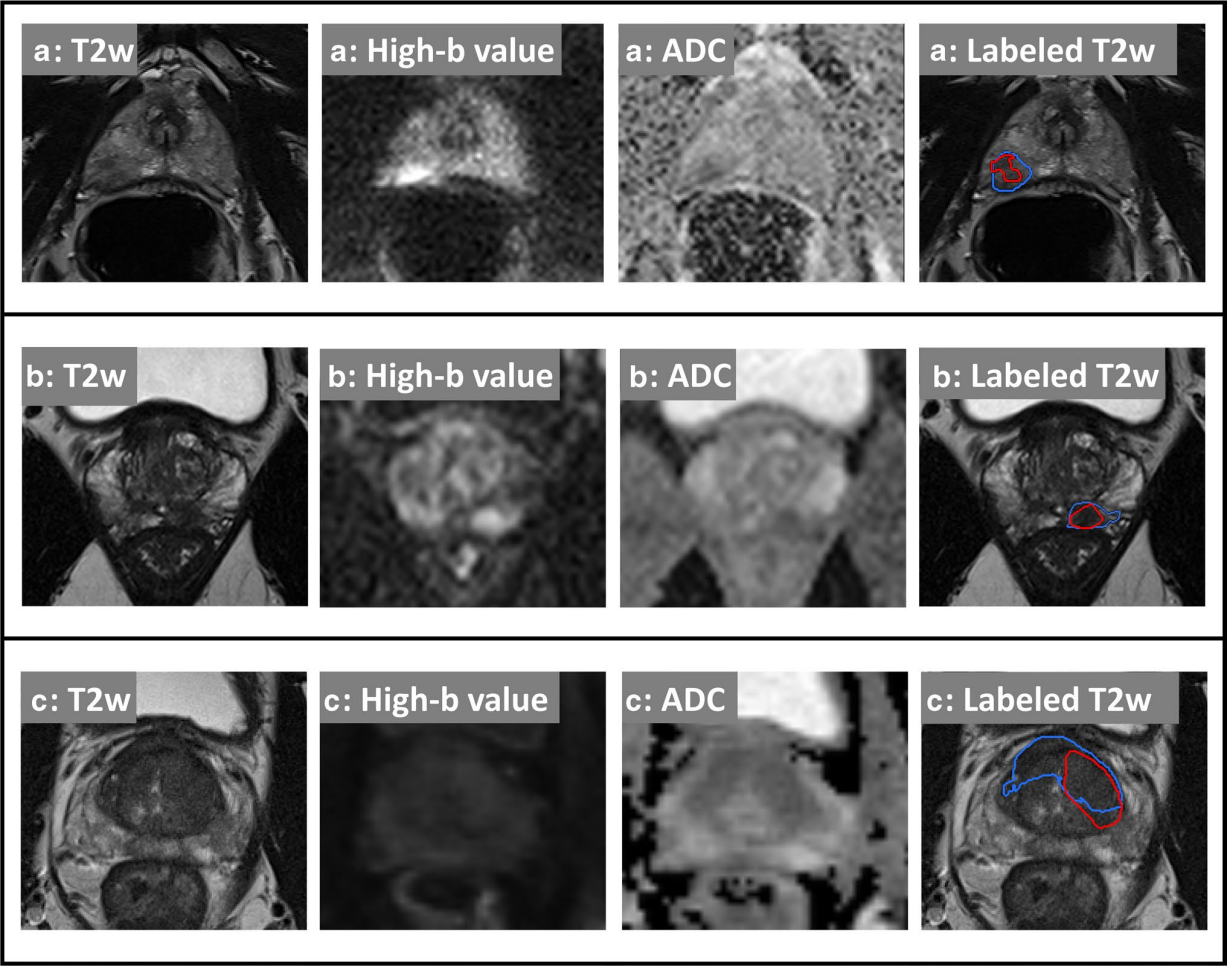
Case examples: from left to right: T2w, DWI high *b*-value, ADC map, and overlay of T2w with radiologist (red) and UNETM segmentation (blue) shown at a threshold corresponding to a sensitivity ≥ 95% in patient-based analysis. Cases from Düsseldorf (**a**), Heidelberg (**b**), and PROSTATEx (**c**) are shown. The images shown are co-registered and cropped. Upper row: 74-year-old patient, PSA 5.25 ng/mL. Suspicious 14-mm PI-RADS 4 lesion in the right peripheral zone (PZpl) at the apex, hyperintense on high *b*-value DWI and with moderately reduced ADC (rs-EPI). Targeted biopsy contained no significant carcinoma (ISUP Grade Group 1). This case illustrates the average ITS-Dice with a score of 0.36. Middle row: 63-year-old patient, PSA 6.69 ng/mL. Suspicious 13-mm PI-RADS 4 lesion in the left peripheral zone (PZpm) at mid to base with hyperintensity on high *b*-value DWI and focally reduced ADC (ss-EPI) was jointly detected by UNET and radiologists with an ITS-Dice of 0.64. Targeted biopsy revealed 20% ISUP Grade 2 carcinoma in 3/5 cores. Subsequent prostatectomy confirmed T3a disease with microscopic extracapsular extension with a Gleason score of 7a. Lower row: 66-year-old patient with a large, homogeneously T2-hypointense mid anterior transition zone (TZ) lesion with reduced ADC (ss-EPI). Biopsy revealed clinically significant carcinoma (ISUP Grade Group ≥ 2). UNETM and the human reader had moderate spatial agreement with ITS-Dice of 0.43

**Fig. 3:**
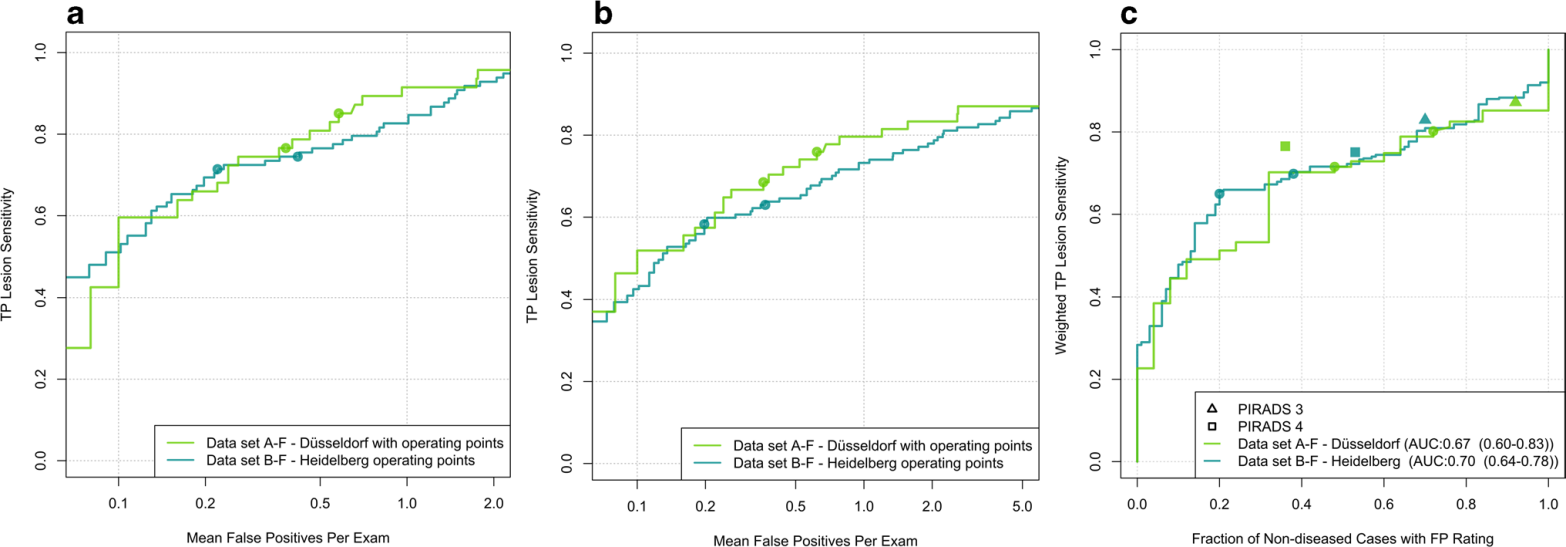
Lesion-level analysis in cohorts A-F (green) and B-F (cyan). CNN operating points at 90% and 95% patient-level sensitivity are indicated as dots. **a** FROC curves using systematic-enhanced lesion ground truth as reference (MRI lesions with csPCa proven either in corresponding matched biopsy cores or in systematic biopsy cores of overlapping sextants). **b** FROC curves obtained using sPC-positive sextant-extended ground truth as reference (the ground truth from (A) extended by sextant segmentations of lesion-free sextants with csPCa demonstrated in corresponding systematic biopsy cores). **c** waFROC analysis obtained using sPC-positive sextant-extended ground truth. For comparison, clinical PI-RADS performance is indicated as triangles (PI-RADS 3) and circles (PI-RADS 4)

**Fig. 4:**
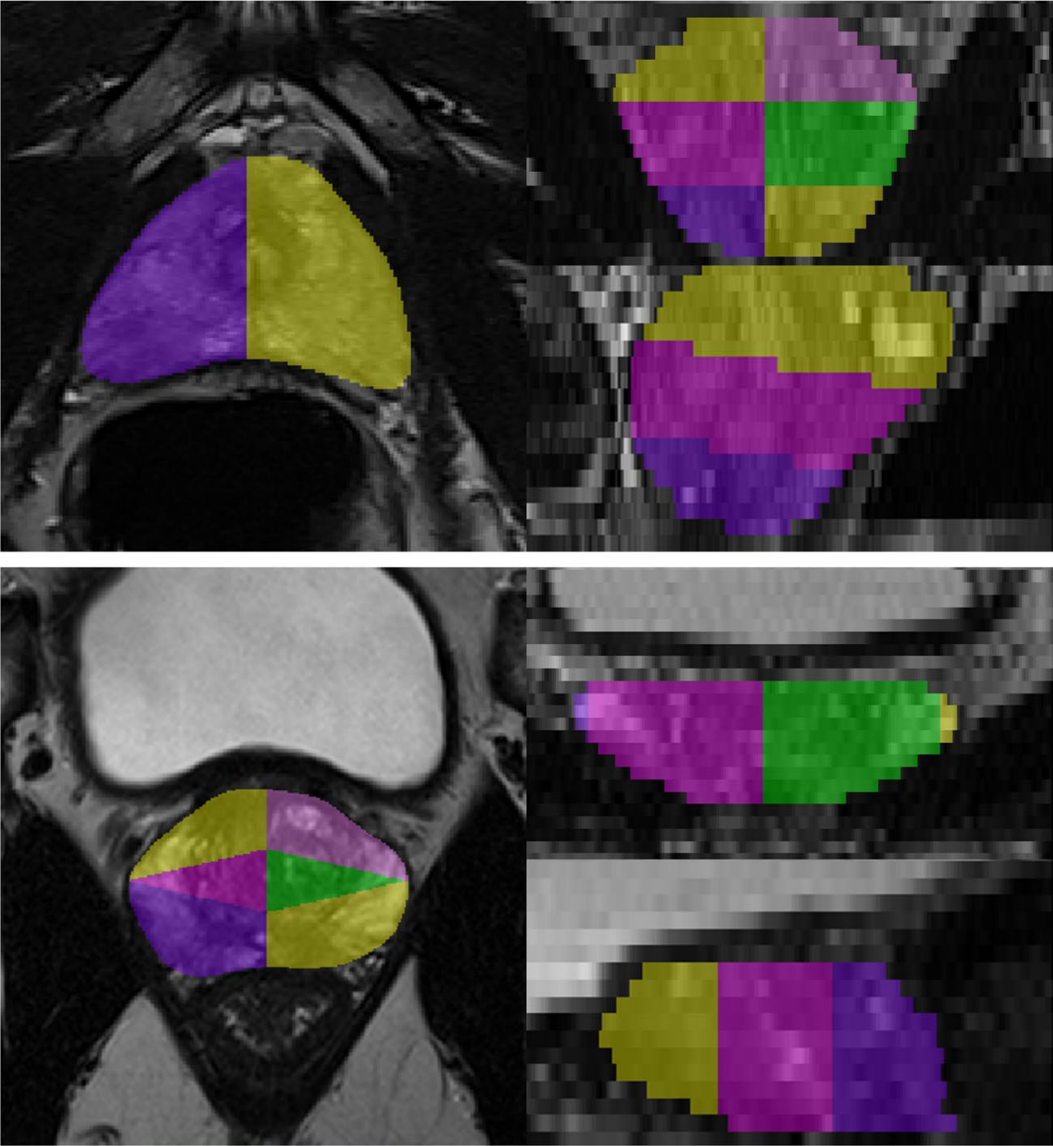
Sextant parcellation schemes used in Düsseldorf (top) and Heidelberg (bottom) to model clinical biopsy strategies (transrectal and -perineal, respectively). Note the angulated planes used to accurately model transrectal biopsy

Thirty-eight percent (data set A-F) and 49% (data set B-F) of clinically detected lesions had a PI-RADS score of 4. 25% (A-F) and 17% (B-F) had a PI-RADS score of 5. In data set A-F, the Dice coefficient [25] of jointly detected lesions (intent-to-segment, ITS) was 0.36 between CNN and manual segmentations. In data set B-F, lesions had a mean ITS-Dice coefficient of 0.49 between CNN and manual segmentations. In data set C, the mean ITS-Dice coefficient between CNN and manual segmentations was 0.43. Examples of obtained lesion segmentations for all data sets are shown in Fig. 2. Figure 3a and b show FROC analysis. At 1 false positive per exam, CNN lesion sensitivity for radiologist ground truth (3A) in data set A-F is 92% (43/47) and 83% (81/98) in dataset B-F. Using fusion ground truth (3B), CNN sensitivity is 80% (43/54) and 73% (93/127), respectively. Figure 3 C shows CNN waFROC analysis with an AUC in data set A-F of 0.67 (95% CI 0.6–0.83) and 0.7 (95% CI 0.64–0.78) in data set B-F. In both data sets, the CNN reaches performance comparable radiologist assessment. Table 4 shows performance at patient-level operating points. Figure 4 shows the sextant parcellation in data set A and B used for fusion ground truth.

**Table 4:** Lesion-level assessment of CNN performance at patient-level thresholds

Patient-level sensitivity	Data set	Lesion-level sensitivity	Weighted lesion-level sensitivity	Mean FP/exam	Fraction of FP non-diseased cases
Radiologist ground truth					
0.90	A-F	0.77 (36/47)	0.75	0.38	0.42 (11/26)
0.90	B-F	0.71 (70/98)	0.74	0.22	0.22 (23/104)
0.95	A-F	0.85 (40/47)	0.87	0.58	0.58 (15/26)
0.95	B-F	0.75 (73/98)	0.77	0.42	0.44 (46/104)
Fusion ground truth					
0.90	A-F	0.69 (37/54)	0.72	0.36	0.48 (12/25)
0.90	B-F	0.58 (74/127)	0.65	0.2	0.20 (20/100)
0.95	A-F	0.76 (41/54)	0.80	0.62	0.72 (18/25)
0.95	B-F	0.63 (80/127)	0.70	0.37	0.38 (38/100)

### Calibration thresholds in different data sets

**Fig. 5:**
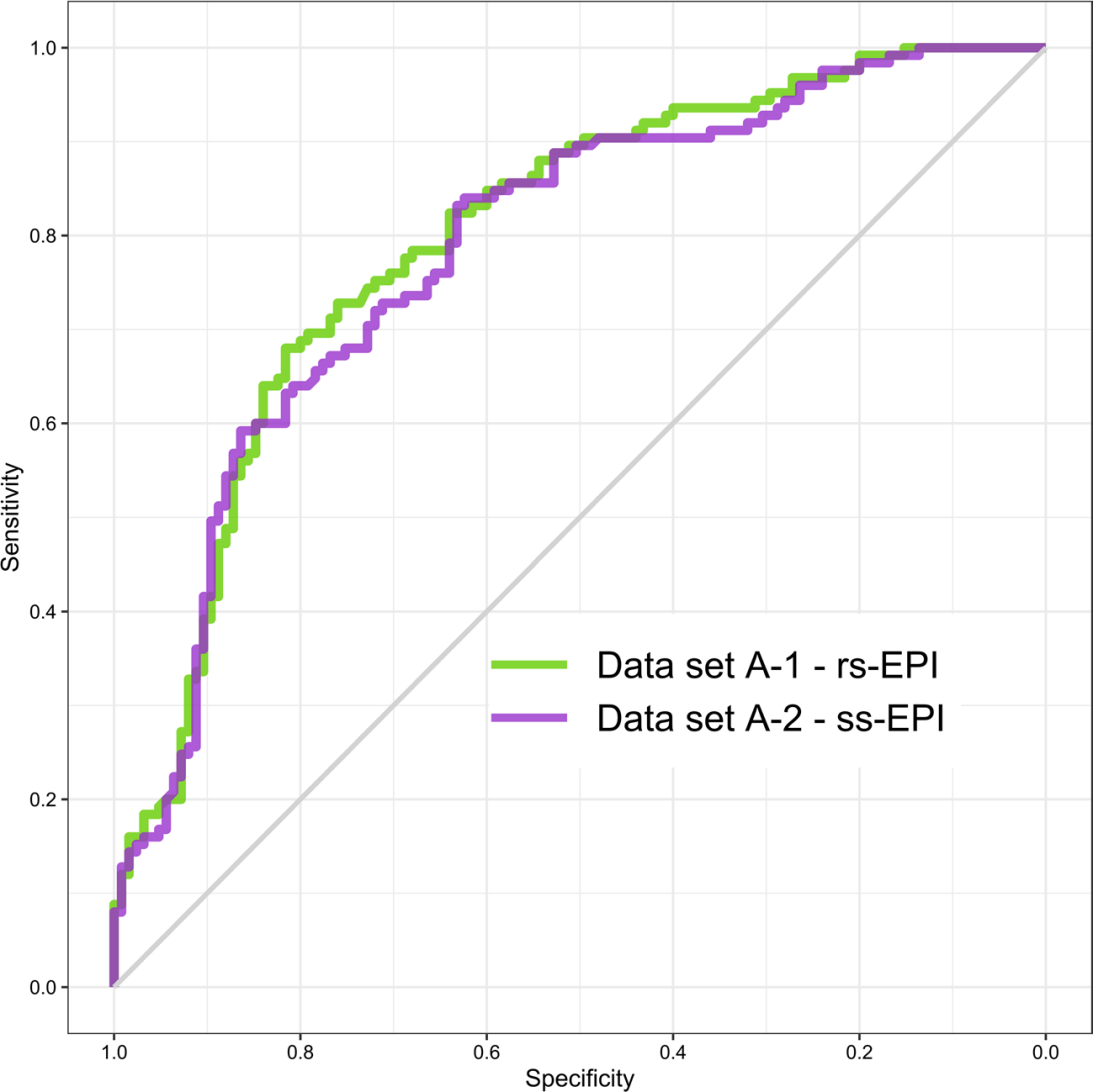
ROC curves showing patient-level performance on different diffusion sequences available for the same 250 examinations in Düsseldorf. Using identical T2-weighted images, the cohorts differ only in the type of diffusion imaging chosen for each case. Data set A-1 (green) is composed of readout-segmented echo-planar imaging (rs-EPI) whenever available (173/250), while data set A-2 (cyan) places emphasis on single-shot echo-planar imaging (ss-EPI) (178/250). The area under the receiver operating characteristics curve was slightly higher when rs-EPI imaging was preferred with 0.80 compared to 0.79 when ss-EPI imaging was used primarily

Applying softmax thresholds gained from calibrating to ≥ 90%/95% sensitivity in each data set to the other data sets shows drifting operating points (Table 5). The 90% sensitivity target shows performance ranging from 74 to 97% depending on data set with specificities ranging from 37 to 76%. Absolute threshold values range from 0.08 to 0.18. The 95% sensitivity target shows performance ranging from 89 to 100% with specificities ranging from 7 to 50% and threshold values from 0.04 to 0.09. Comparing patient-level ROC performance when varying diffusion sequence in data sets A-1 and A-2 (Fig. 5), ROC AUC was marginally higher in data set A-1 with 0.80 compared to 0.79 in data set A-2 (*p* = 0.504), with only minor, insignificant differences in operating points.

**Table 5:** Performance using thresholds calculated using data from different institutions. Operating points show a significant drift across different data sets when thresholds are not recalibrated. Bold numerals indicate that the specific threashold was calibrated within the corresponding cohort

Calibration set	Threshold	Sens./spec. data set A-1	Sens./spec. data set A-2	Sens./spec. data set B	Sens./spec. data set C
A-1 ≥ 0.90 sens.	0.12	**0.90/0.50**	0.86/0.58	0.93/0.59	0.87/0.50
A-2 ≥ 0.90 sens.	0.08	0.94/0.39	**0.90/0.48**	0.95/0.46	0.97/0.37
B ≥ 0.90 sens.	0.18	0.83/0.6	0.74/0.66	**0.91/0.71**	0.76/0.76
C ≥ 0.90 sens.	0.11	0.90/0.46	0.86/0.56	0.94/0.56	**0.91/0.49**
A-1 ≥ 0.95 sens.	0.06	**0.95/0.3**	0.91/0.36	0.96/0.41	0.99/0.19
A-2 ≥ 0.95 sens.	0.04	0.98/0.2	**0.96/0.26**	0.97/0.28	1.0/0.07
B ≥ 0.95 sens.	0.07	0.94/0.31	0.9/0.39	**0.96/0.44**	0.97/0.22
C ≥ 0.95 sens.	0.09	0.92/0.42	0.89/0.5	0.95/0.47	**0.97/0.43**

## Discussion

For physicians and vendors of AI systems, an important question is whether an AI system validated and trained using data from one or few institutions can be applied without change within their specific practice setting. Our data further confirm that at least in the given single-vendor setting, a large amount of diagnostically decisive information can be extracted from bi-parametric prostate MRI from external independent practice even without re-training a foreign model. These findings are encouraging as they suggest that at least workable starting conditions can be established without prior training of CNN models at new practice sites wishing to establish AI methods.

Importantly, application of the model requires re-calibration of the probability thresholds to the new environments. It has been previously noted that re-calibration should follow in regular quality-control cycles [3], as even within one institution protocol changes or referral patterns can lead to performance changes over time. The latter is demonstrated in the datasets A-1 and A-2, in which a different diffusion sequence was used, requiring calibration adjustment. When moving between institutions our data show that this effect becomes even more pronounced, resulting in clinically unacceptable operating points if thresholds were to be applied without recalibration at the target institution.

While the decrease in overall discriminative ability of our model on the external test sets did not reach statistical significance as previously reported in brain tumor segmentation by AlBadawy et al [11] and in PET/MRI enhancement by Chen et al [10], our findings are in line with reports of slight degradation in CT head and neck squamous cell carcinoma classification by Kann et al [9] and histopathological polyp classification by Wei et al [26]. The examined DL system achieved significantly better specificity within the training institution for the ≥ 0.90 sensitivity threshold, indicating that some performance is lost during transfer that may be correctable with re-training. Despite the small observed performance loss, CNN performance in all of the examined balanced data sets lies within the performance range reported for clinical PI-RADS assessment [27], as far as can be determined from the artificially balanced non-consecutive datasets. The balanced design, however, allows to closely compare the performance of a DL system that has been established and validated in data from consecutive practice previously on unseen external datasets without the confounder of different lesion characteristics in the cohorts, allowing us to attribute the observed performance loss to the properties of the images and the reporting style and mitigating the influence of different prevalence.

In accordance with the higher specificity observed at a sensitivity level of 0.90 within the internal cohort B in the exam-level analysis compared to cohort A, lesion-level analysis demonstrates a lower number of mean false-positives per exam in cohort B at both the exam-level 0.90 and 0.95 cutoffs. Interestingly, however, this occurs at a lower lesion-based sensitivity for cohort B compared to cohort A, while exam-based sensitivity remains fixed at 0.90 or 0.95. This finding of achieving an identical exam-level sensitivity through a lower lesion-based sensitivity in cohort B is likely due to different reading styles at both institutions. In the case of multiple lesions, the non-index lesions are more likely to be less apparent at institution B that thus harbor sPC less frequently and are often replaceable by the index-lesion with regard to the overall exam-level diagnosis. Thus, a lower fraction of the individual lesions require to be correctly identified by DL to achieve the same exam-level sensitivity as in cohort A. Lesion-level analysis is important for assessing the performance of AI systems [28], which however requires to be considered in the context of the exam-level diagnosis. The Dice-score itself does not necessarily determine successful lesion detection, as different raters often agree on at least a portion of a lesion, as reported with Dice-scores of 0.48–0.52 [29]. Thus, while lesion boundaries from different raters vary, the presence of sufficient overlap even at a lower Dice-score may be as diagnostically valuable as complete overlap. The observation of lower Dice-scores in cohort A-F is most likely the result of a different outlining style of the rater that annotated this data set, as opposed to the raters that outlined the training data at institution A. From the data, it is both possible that the lower Dice-overlap in cohort A contributed to the necessity to lower the probability threshold of the DL system, thus increasing the number of false positives, or that the properties of the images were different enough in cohort A, such that the DL algorithm trained at institution B had difficulties in establishing distinctions as well as in data from the training institution. FROC analysis carries its own limitations. While application of FROC analysis in prostatectomy cohorts [30, 31] is based on a more clearly defined gold standard, application in biopsy cohorts [32, 33] suffers from limited histopathological information about regions outside of MRI-visible lesions. Future studies need to carefully report how this performance is assessed as impact is measurable. Comparing FROC analyses based on radiologist ground truth alone to fusion ground truth reveals that relying on the former leads to overestimation of lesion-level performance, as all lesion-based sensitivities decrease. The observed decrease in false-positives per exam is explained by assumedly false positive lesions being re-classified as true positives based on the sextant scheme and systematic biopsies. These lesions are more likely to carry more subtle imaging characteristics, e.g., PI-RADS 3, and are more difficult to detect by DL at probability thresholds that preserve specificity. The increasing gap between cohort A and B in relationship to lesion-level sensitivity when comparing radiologist vs. fusion ground truth is likely attributed to the more extensive systematic biopsy scheme at institution B, discovering more sPC outside of the index lesions. The waFROC analysis is able to correct for these differences successfully, by providing a means to emphasize the importance of sPC-negative cases at the exam level. Thus, while the general appearance of the FROC curves is that in cohort A, a higher sensitivity is achieved at the same false positive rate, this does not indicate superior lesion-level performance, as there are on average higher PI-RADS score MR lesions called in cohort A. Thus, the operating points in cohort A are shifted toward the right, resulting in higher sensitivity at the same exam-level sensitivity. The combination of more than double the number of systematic biopsies plus more MR targets per patient in cohort B leads to a higher likelihood that small cancers are diagnosed, and artificially lowers the apparent sensitivity in standard FROC analysis, requiring waFROC analysis to correct for the influence of more and subtler lesions per exam in cohort B. As these findings show, FROC analyses need to be carefully interpreted in the context of the underlying cohort. These comparative findings would not be possible to discern in non-balanced cohorts from prevalence effects, supporting the importance of the chosen study design for focusing on comparison on discriminative performance in different cohorts.

While the difference between data sets A-1 and A-2 was only slight, it is interesting that the model seems to favor the rs-EPI emphasized data set A-1 instead of the EPI sequence seen during training and in data set A-2. This could be the result of improved image quality of rs-EPI [34] and a reduction of gas-induced susceptibility artifacts [35], leading to—empirically observed—superior co-registration.

Given the similar performance of the UNETM model in the two external cohorts, it appears that the domain of prostate MRI is well-defined enough on a large-enough prostate MRI data set from multiple scanners such that external same vendor datasets do not appear to present a true domain change for the model. Rather, the variability, at least in the two examined external cohorts, appears to be controlled enough that no unexpected noise patterns or image textures were encountered to severely affect the model performance. Nevertheless, while the amount of transferable performance appears to be substantial, there remains uncertainty about the full range of conditions that may affect CNN performance in external settings, given the still small number of external sites and the small amount of data evaluated. This is especially true in the context of prostate imaging, where lack of adherence to PI-RADSv2.1 criteria remains common [36, 37] and has the potential to affect diagnostic performance [38]. Thus, continued study of transferability of established models in use settings is required to further support applicability of models in new environments.

Using cohorts balanced with regard to csPCa and non-csPCa examples or PI-RADS scores, CNN evaluation can be focused on direct comparison of discrimination ability between institutions, and thus isolate the decision problem, as it does not allow the machine learning algorithm to adjust decision based on local factors such as different prevalence of different referral patterns. At the same time, the balanced cohort construction has the disadvantage of not providing the actual prevalence of csPCa in typical consecutive clinical practice, which has been shown to lie at around 15–38% [6, 39]. The comparison between institutions is homogenized by this approach, as differences in prevalence or PI-RADS distribution that would exist when comparing consecutive clinical practice would affect model performance. Still, differences in tumor biology between practices can still play a role.

There are limitations to our study: While demonstrating transferability of an institutional model to two external data sets, this concept requires to be expanded to larger multi-centric settings in the future to confirm findings. Voxel-wise annotation and correlation with targeted biopsies is, however, very time intensive. If CNN generated segmentations are to be used to target biopsies, further examination of overlap and differences to radiologist segmentations is needed. In this single vendor setting, MRI protocols used in cohorts A and B were similar and no DCE-MRI was used. Cohort C has known issues in terms of the imaging protocol, which however provides a reference to a popular public domain challenge data set, with the comparable DL performance to external data set A underlining its usefulness despite its limitations [40]. Evaluation was retrospective and focused on constructed data sets for reasons of comparability. Evaluation of CNN performance in prospective studies and in consecutive practice is required in future studies in comparison to board-certified radiologist PI-RADS and AI-supported performance to confirm and assess that AI leads to measurable improvements in clinical workflows, diagnostic quality, and efficiency. Protocols during the study period were not in full agreement with PI-RADS and PI-QUAL [41] recommendations; i.e., of the two centers A and B, protocols at center B used larger FOV than recommended for the DWI sequence. Importantly, voxel size was within the recommended limits, providing optimal image quality at high resolution and SNR. As an experienced center in prostate MRI, center B constantly reviews image quality and had decided to use larger FOV for improved SNR for DWI, while the rationale for PI-RADS to recommend a smaller FOV is primarily to focus image optimization to the prostate and assure sufficient resolution. Importantly, according to PI-QUAL, a minor protocol deviation would still be considered a fully diagnostic examination, e.g., lowering an excellent study from PI-QUAL 5 to 4. Balancing the data results in cohorts that differ in composition from clinical routine potentially introducing bias. However, the approach chosen results in balanced data sets with regard to the obviousness of the index lesion, thus allowing to examine DL performance independently from cohort characteristics for better comparability and to focus on DL discriminative performance itself. Importantly, the evaluated DL model has been developed and validated on consecutive clinical data and its performance in that setting is known. The current study design is optimized toward evaluating differences in discriminative ability independent of prevalence. All patients included received biopsies, which could have introduced bias. However, at both institutions A and B, primary focus was to select a balanced number of consecutive MR examinations with positive and negative histopathology. As such, the AI is challenged with typical presentations of difficult decisions between histopathologically benign and malignant lesions. An inclusion of primarily very low suspicion MRIs would not have supported our aims similarly. Annotations were performed by multiple raters; however, all raters based their annotations on written clinical reports and their accompanying diagrams and annotations were reviewed by experienced radiologists, to reduce variability as much as possible.

In conclusion, in this study, a deep learning system for fully automatic detection and segmentation of lesions suspicious for clinically significant prostate cancer previously trained with mono-centric multi-scanner data and validated on bi-centric multi-scanner data demonstrated transfer capability in a single-vendor situation on independent external data sets from different institutions despite absence of training examples from these, indicating that a large amount of clinically pertinent information can be extracted even from data originating from an institution not included in training and validation.

## Abbreviations

CNN: Convolutional neural network
csPCa: Clinically significant prostate cancer
PI-RADS: Prostate Imaging Reporting and Data System
T2w: T2-weighted
ISUP: International Society of Urological Pathology
PSA: Prostate-specific antigen
DL: Deep learning
EPI: Echo-planar imaging
rs-EPI: Readout segmented EPI
ss-EPI: Single-shot EPI
TRUS: Transrectal ultrasound guided prostate biopsy

## Declarations

### Guarantor

The scientific guarantor of this publication is David Bonekamp.

### Statistics and biometry

Thomas Hielscher is lead statistician and co-author on this paper.

### Study subjects or cohorts overlap

The Heidelberg data set is a subset of a previously published test cohort for evaluation of CNN-models including the TD-A model [1]. Part of the Düsseldorf data have been previously included in a study about reasons for missing sPC on targeted biopsy [2].

### Methodology

• retrospective

• diagnostic or prognostic study

• multi-center study
